# Genome Sequences of Two Multidrug-Resistant *Acinetobacter baumannii* Clinical Strains Isolated from Southern India

**DOI:** 10.1128/genomeA.01010-15

**Published:** 2015-09-10

**Authors:** Veeraraghavan Balaji, Sangeetha Rajenderan, Shalini Anandan, Indranil Biswas

**Affiliations:** a Department of Clinical Microbiology, Christian Medical College, Vellore, Tamil Nadu, India; b Department of Microbiology, Molecular Genetics and Immunology, University of Kansas Medical Center, Kansas City, Kansas, USA

## Abstract

*Acinetobacter baumannii* is an emerging nosocomial pathogen causing infections worldwide. In this study, we determined the genome sequences of two multidrug-resistant *A. baumannii* clinical strains isolated from a hospital in southern India. Genome analyses indicate that both the strains harbor numerous horizontally transferred genetic elements and antibiotic resistance cassettes.

## GENOME ANNOUNCEMENT

*Acinetobacter baumannii*, a Gram-negative nonfermenting aerobic bacterium, is responsible for a variety of infections, including bacteremia, meningitis, skin and soft tissue infections, and ventilator-associated pneumonia (1). This pathogen has now become a major causative agent of nosocomial infections worldwide due to its extraordinary ability to acquire novel resistance determinants to numerous antimicrobial compounds (2). The carbapenems are generally used for the treatment of infections caused by multidrug-resistant (MDR) *A. baumannii*; however, in recent years, carbapenem-resistant *A. baumannii* bacteria are also on the rise.

In this report, we determined the draft genomes of MDR *A. baumannii* clinical isolates harboring New Delhi metallo-β-lactamase (NDM) determinants. The first strain, B11911, was isolated from a patient with bloodstream infection, while the second strain, SP1917, was isolated from a patient with ventilator-associated pneumonia.

The B11911 and SP1917 strains were sequenced using P6/R4 chemistry in the PacBio RS II sequencing platform. The sequencing generated 30,578 reads with a mean length of 10,896 bp for B11911 and 17,292 reads with a mean length of 9,742 bp for SP1917 from a single SMRT cell. The sequences were assembled *de novo* with Hierarchical Genome Assembly Process 3 (HGAP3; SMRTAnalysis, version 2.3.0) (3, 4), and the contigs were trimmed with Minimus 2 (5). The draft genome of B11911 consists of 4 contigs encompassing a total of 4,263,915-bp sequence with an *N*_50_ contig size of 4,018,724 bp. The genome assembly suggests the presence of one chromosome (trimmed) and one plasmid (trimmed), and a portion of a small plasmid (untrimmed) in the B11911 genome. The draft genome of SP1917 consists of 10 contigs containing a total sequence of 4,484,902 bp with an *N*_50_ contig size of 2,911,226 bp. The genome assembly indicates the presence of a chromosome (trimmed) and three circular plasmids (untrimmed) in the SP1917 genome. Both genomes were annotated using the Analysis Engine at the University of Maryland (6). Analysis at the CGE server (http://www.cbs.dtu.dk/services) indicates that B11911 belongs to sequence type 149 (Pasteur), while SP1917 belongs to an unknown sequence type. ResFinder-2.1 analysis at CGE server confirms the presence of multiple resistance determinants against aminoglycosides, β-lactams, macrolide (and related antibiotics), chloramphenicol, rifampin, and sulfonamides antibiotics in the genomes. The analysis failed to identify any resistance genes against fluoroquinolone, fosfomycin, fusidic acid, glycopeptides, oxazolidinone, and trimethoprim in the genomes. However, SP1917 appears to encode a tetracycline resistance cassette (*tetB*), but no such resistance genes were found in B11911. As for the β-lactam resistance, SP1917 appears to harbor *bla*_OXA-23_, *bla*_OXA-144_, and *bla*_NDM-1_ alleles, whereas B11911 appears to harbor, in addition to *bla*_OXA-23_ and *bla*_NDM-1_, *bla*_PER-7_, and *bla*_OXA-203_ alleles. In both genomes we identified five putative secondary metabolite gene clusters (including acinetobactin and acinetoferrin) by antiSMASH (7), at least four genomic islands by IslandViewer3 (8), and multiple prophage sequences by PHAST analyses (9). The availability of these two MDR genome sequences will further facilitate comparative genomic analysis and evolutionary studies related to antibiotic resistance mechanisms in *A. baumannii*.

### Nucleotide sequence accession numbers. 

The draft genome sequences have been deposited in DDBJ/EMBL/GenBank under the accession numbers LFYX00000000 (B11911) and LFYW00000000 (SP1917). The versions described in this article are the first versions, LFYX00000000.1 and LFYW00000000.1, respectively.
